# Advocating for the Health Worker

**DOI:** 10.5334/aogh.2461

**Published:** 2019-01-28

**Authors:** Melissa McDiarmid

**Affiliations:** 1 University of Maryland, School of Medicine, Division of Occupational and Environmental Medicine, Baltimore, MD, US

## Abstract

**Background:**

Health workers in both well-resourced and limited income settings face health threats from exposures encountered in their unique and complex work environment. Even before the 2014 Ebola outbreak, preventable harm was routinely felt by health workers, most visibly through the fatal collusion between the HIV/AIDS epidemic and tuberculosis (TB) infection in high endemic countries.

**Objective:**

The aim of this paper is to examine the analyses of the health sector workforce by development and public health agencies regarding its sustainability, threats from workers’ personal health risks and discussion of protections to address those risks.

**Methods:**

Development and public health agency reports assessing the sustainability of and threats to the health workforce both pre-and post the 2014 Ebola outbreak were examined with a focus on low and middle- income countries (LMICs).

**Findings:**

Reviews of the health sector workforce have largely focused on its role as a necessary component of sustainable development. Hence, staff competency, numbers and productivity have been emphasized with little notice of the conditions of work and the highly hazardous environment contributing to worker out-migration, illness and death.

**Conclusions:**

Going forward, the 2016 World Health Assembly campaign to advance human resources for health and other UN efforts on health employment may offer some opportunities to address needed health worker protections. However, to these largely competency-focused workforce development efforts must *first* be brought resources for and commitment to protecting the safety of these workers’ lives and livelihood. Doing less defeats investments in fragile health systems and is plainly unethical.

## Introduction

In both well-resourced settings and in low- and middle-income (LMI) countries, the health workforce is threatened with harm from exposure to agents encountered in a unique and complex work setting.

While the Ebola outbreak is the most recent example of preventable harm faced by the health workforce [1], serious threats to these workers’ lives and livelihoods predated that historic devastation, most visibly through the HIV/AIDS epidemic and the fatal collusion between HIV/AIDS and tuberculosis (TB) infection in high endemic HIV regions [2]. Such outcomes cripple health systems in affected areas, further jeopardizing capacity to prevent and treat wider advancing health threats, both infectious and otherwise.

Given these sobering and on-going risks to both the work force and the larger health system, worker protections must take on a greater urgency than previously afforded by health ministries, sector planners and donors alike.

## Unique Hazards and Challenges of the Health Sector

The health sector is best identified with the risk of exposure to life-threatening infections such as TB, SARS, HIV, and hepatitis, which have been the primary focus of health worker (HW) safety programs, where they exist. Also historically recognized were cancer excesses in radiology personnel which have declined with safety interventions [3]. More recently acknowledged have been the chemical hazards, including novel agents, some unique to the sector such as sterilants, laboratory reagents, and pharmaceuticals including the highly toxic anti-cancer drugs [4]. Also recognized are musculo-skeletal injuries resulting from patient lifting and workplace violence against health workers, which have contributed to poor job conditions and staff dissatisfaction [5].

The altruistic, caregiving mission of the sector also complicates prevention practices because typical workplace self- preservation behaviors are suspended in a culture of selfless commitment to patient care. There is an erroneous ‘either/or’ mentality often present that sometimes forces a worker to make a false choice between providing good care or protecting one’s own health [6].

Conditions of work and threats to health have been found to contribute to the current global HW shortage [7] and even in well-resourced settings, are critical factors in job satisfaction and nurses’ decisions to stay in the profession [8].

## Health Worker Protections Agenda

While the vulnerabilities of the global health workforce have been well known for more than twenty years, consistent, systematic protection efforts by public health and development agencies have been disappointingly lacking.

An early reference to HW safety was made in a World Health Assembly (WHA) resolution, titled “Global Strategy for Occupational Health [9]” where, the “complex combination of hazards” in the health sector was expressly called out as requiring attention.

This resolution pre-dated the spate of international development agency reports that would follow in the late 1990s through the mid- 2000s describing the toll the HIV/AIDS epidemic was taking on health systems in LMI countries, especially in sub-Saharan Africa [2].

The connection between HIV infection and TB susceptibility was sufficiently well documented by the late 1990s that the WHO issued a policy statement recommending preventive isoniazid therapy to persons living with HIV [10]. It would take more than an additional decade for that and other preventive policies to be specifically recommended for the health worker living with HIV [11], despite the evidence that TB risk in health workers is “consistently higher” than risk among the general population world- wide [12,13,14].

Importantly, most of the analyses of the health sector by development agencies was performed from an economic development vantage point, viewing the workforce as an element of the broader health system hobbled by the burden of the epidemic generally and less so as a targeted victim of it.

Certainly, this emphasis was understandable given these agencies’ mission focus on sustainable development and view of the health system as a necessary component toward that end. Thus, the attention to numbers of workers, capacity, competency, productivity, and worker to population ratios were often the metrics used to describe the health workforce for these reports [15].

Despite the presence of data to the contrary [13,16], health worker absenteeism, illness, and death were largely ascribed to workers being members of the wider, general population affected by the HIV epidemic. Typically, not appreciated was the risk from providing care to affected patients, often without safety training, prescribed work practices, protective vaccinations, and proper equipment.

Acknowledging the urgency of the HW crisis, explicit mention of “worker illness and death” as one aspect of HW “out-migration” from the workforce was made in the 2006 World Health Report, “Working Together for Health”, in which the WHO warned that, “losing its workforce can bring a fragile health system close to collapse [6]” and urged improvements in “conditions of work” in the health sector.

The health workforce was also promoted as one of six essential “pillars” of WHO’s health system strengthening initiative in 2010 [17]. While its focus was again on raising worker numbers and clinical capacity it also targeted for intervention workforce shortages and “losses caused by death, retirement, career change, or out migration”.

Reinforcing this health workforce development campaign, the World Health Assembly (WHA) in 2016, approved a WHO proposal advancing a global strategy for human resources for health (HRH) through 2030 [18]. The proposal however, is disappointingly sparse on any mention of occupational health protections for these workers. Though the concept is mentioned, it contains no specifics even though detailed specifics are included regarding education and training goals and skills acquisition.

Some of the worker- focused sustainable development (SD) goals adopted at the 2015 UN SD Summit [19] might have been woven in here, including providing for the social protection and surveillance of high-risk workers and promoting the International Labor Office (ILO) campaign of “decent work” [20], a principal tenant of which is to provide a “safe working environment”.

Fortunately, the notion of ‘decent work’ was included in the UN High-Level Commission on Health Employment and Economic Growth convened in 2016 to address the global HW shortage through cross-sectoral collaborations [21] which may promote safety assurances for HWs not currently provided them.

## What will keep health workers reporting to duty?

In the late fall of 2014, during the Ebola outbreak, a senior official in the U.S. Department of Health asked a panel of occupational health and infection control experts, “What will make medical workers and responders feel safe enough to keep reporting for duty?” This question was asked because some U.S. workers failed to report for duty because of fear for their own safety and their lack of confidence in safety preparedness of their employers.

The health worker’s ethical “duty to (provide) care” during pandemics and public health emergencies has been raised previously after similar events and in the literature, without resolution. The challenge arises in the need to clarify and balance the extent and limits of the health worker’s ethical duty to provide care, on the one hand, with the employing organization’s ethical responsibility to protect the workforce by providing safety measures, on the other [22,23]. Given that many organizations’ safety efforts during the Ebola pandemic fell woefully short, both in LMI and developed countries, this balancing of ethical duties and responsibilities remains unresolved.

We know however, from occupational health research that when the worker is confident of the employer’s organizational commitment to safety, they will continue to report for duty. This has been shown by measuring safety culture confidence, one example of which is the availability and quality of personal protective equipment (PPE) [24]. Obviously, a safety program must be more comprehensive than this single aspect, but PPE availability has been viewed as a tangible measure of employer commitment to affected workers.

Expectations that employers, including Ministries of Health, are obliged to provide safe employment for their workers, is not a new idea. Contained in a 1981 ILO Occupational Health Convention, ratified by more than 65 countries, many of which are LMICs, was a provision which required employers to ensure that workplaces were “safe and without risk to health” as reasonably practicable including the control of “chemical, physical and biological substances and agents… [25]”.

More specific to the health sector, the International Commission on Occupational Health (ICOH), in its 2004 document “Recommendations for Protecting Healthcare Workers’ Health”, called for a “systematic occupational risk prevention program” for health workers to include training regarding work risks and the provision of protective measures, as an integral part of an administrative process addressing healthcare quality [26].

These protective measures are specified in basic occupational hazard control methodology and have achieved success in hazard mitigation across many industrial sectors globally [27]. In most cases, these measures can be effectively applied to the health sector [28], even in low resource settings [29].

## Conclusion

In the wake of the bitter lessons of Ebola and the more protracted threat to the health workforce posed by HIV/TB complicity, public health leaders must more deliberately prioritize the safety of workers and improved conditions of work in the health sector. Occupational health expertise and services must be routinized not only in disaster responses but in the usual organization of health services provision. To the competency-focused workforce development efforts currently proposed for the health sector must *first* be brought sufficient resources for and and avid commitment to ensuring the safety of these workers’ lives and livelihoods. To continue to do otherwise propagates “a special cause of wastage” in the health workforce [30], defeats investments in fragile health systems and is plainly unethical.
